# Supplementary material for: Tumor-produced and aging-associated oncometabolite methylmalonic acid promotes cancer-associated fibroblast activation to drive metastatic progression
Source: Nat Commun. 2022 Oct 20;13:6239. doi: 10.1038/s41467-022-33862-0 (PMC9584945; doi:10.1038/s41467-022-33862-0)
Supplement: Supplementary file 1 — Supplementary information [file 41467_2022_33862_MOESM1_ESM.pdf]

Supplementary information

S Figure 1

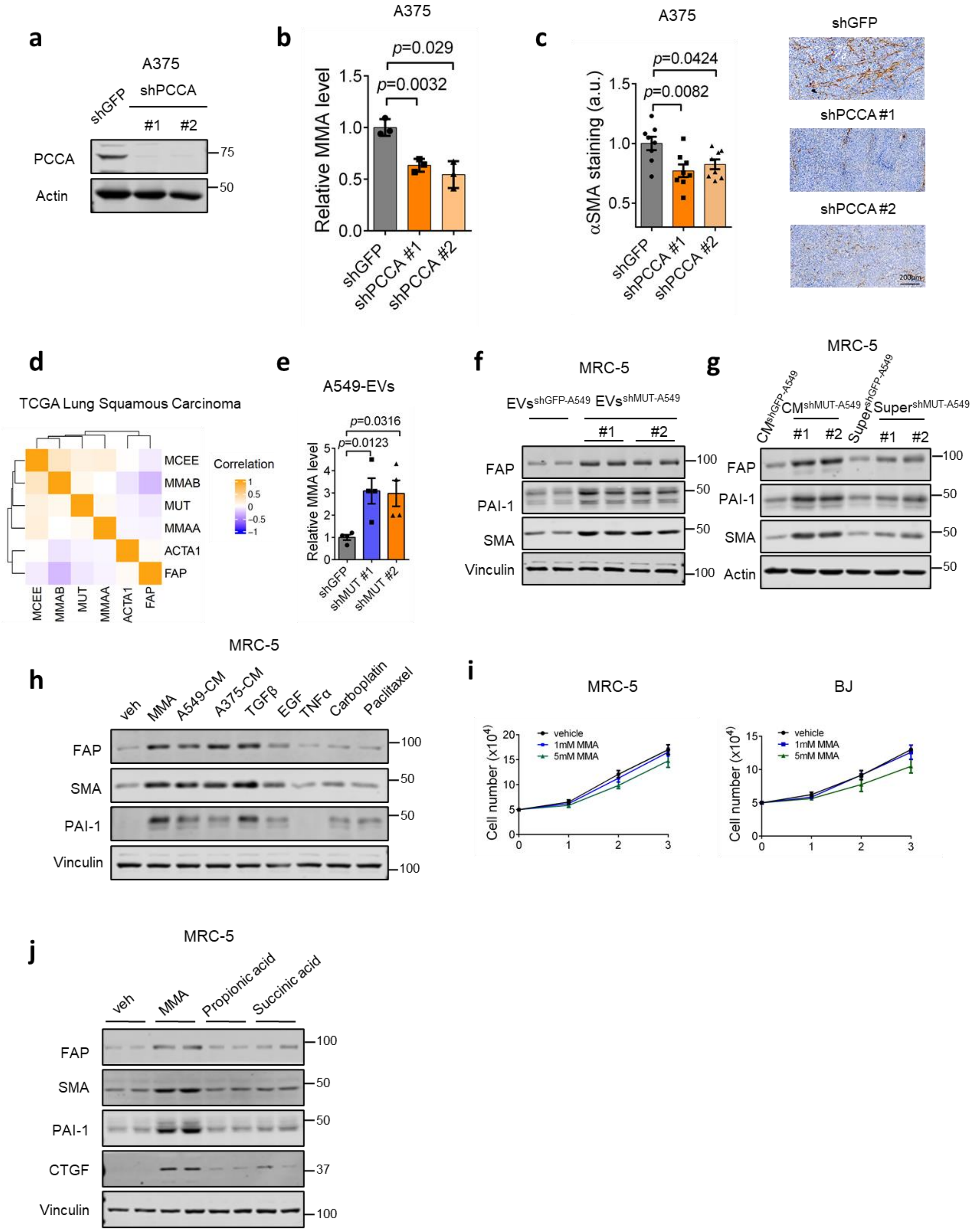

Supplementary Figure 1. MMA promotes CAF marker expression. **a**, **b.** Immunoblots (**a**) and intracellular MMA levels (n=3 independent experiments, mean ± SEM, two-sided paired t-test) (**b**) of A375 cells with sh*GFP* or sh*PCCA* knockdown. **c**. Fibroblast activation levels in tumors of mice xenografted with A375 tumor cells with sh*GFP* or sh*PCCA* knockdown were injected subcutaneously, measured by immunohistochemistry staining of αSMA (n=7-8 independent experiments, mean ± SEM, two-sided paired t-test). **d**. Correlation analysis of an RNA-sequencing dataset of 501 whole tumors from patient lung squamous cell carcinomas showing association between MUT, MCEE, MMAA, MMAB levels and expression of cancer-associated fibroblast markers ACTA1 and FAP. **e**. Relative MMA levels in EVs from A375 tumor cells were measured, normalized to total protein (n=4 independent experiments, mean ± SEM, two-sided paired t-test). **f**. Immunoblots of MRC-5 fibroblasts treated with EVs isolated from sh*GFP* or sh*MUT* A549 tumor cells for 5 days. **g**. Immunoblots of MRC-5 fibroblasts treated with conditioned medium of sh*GFP* or sh*MUT* A549 tumor cells, or with supernatant after removing the EVs, for 5 days. **h**. Immunoblots of MRC-5 fibroblasts treated with 1mM MMA, tumor cell-conditioned medium, 5ng/ml TGFβ, 5ng/ml EGF, 5ng/ml TNFα, 20μM carboplatin or 5nM paclitaxel for 5 days. **i**. Cell proliferation of MRC-5 and BJ fibroblasts after 3 days of MMA treatment (n=3 independent experiments, mean ± SEM, two-way ANOVA). **j**. Immunoblots of MRC-5 cell treated with 1mM MMA, propionic acid or succinic acid for 5 days.

S Figure 2

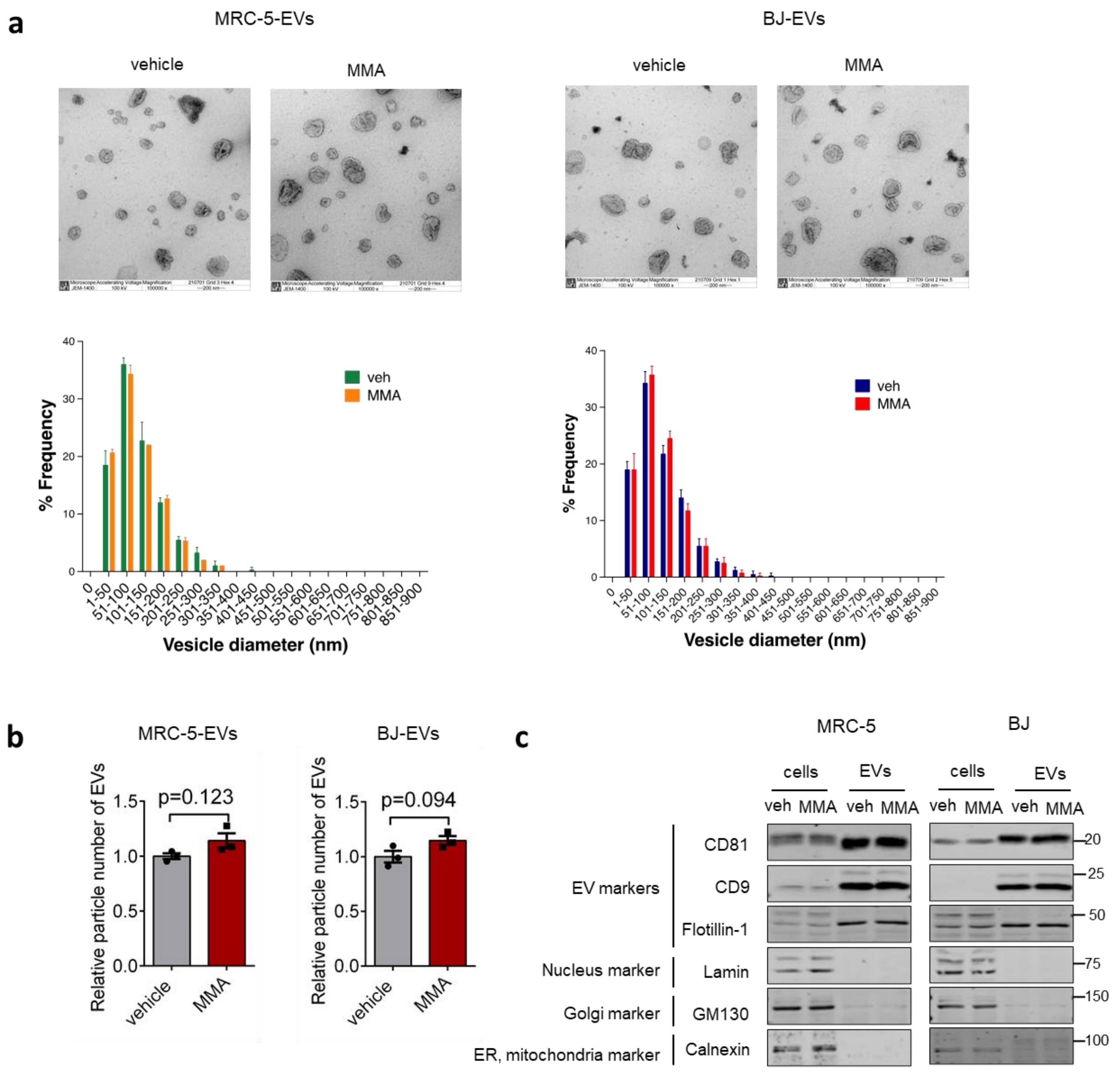

Supplementary Figure 2. **MMA does not change the number or size distribution of EVs.** **a.** Representative electronic microscopy images of extracellular vesicles (EVs) isolated from MRC-5 and BJ fibroblasts after treatment with MMA for 5 days, scale = 200 nm. Statistical analysis of particle size distribution was obtained from n = 1973 MRC-5 veh EVs, n = 1630 MRC-5 MMA EVs n=1939 BJ veh EVs and n = 2459 BJ MMA EVs. (n=3 independent experiments, mean ± SEM, two-sided paired t-test). **b**. Particle number of EVs were measured by NanoSight (n=3 independent experiments, two-sided paired t-test). **c**. Immunoblots of MRC-5 and BJ cell lysate and corresponding EV lysate showing protein levels of typical markers of EVs and different cell components.

S Figure 3

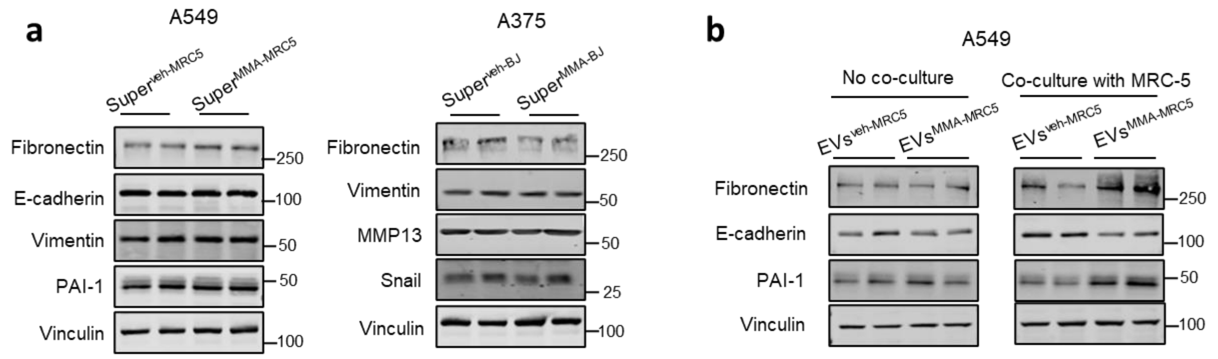

Supplementary Figure 3. Tumor cells undergo EMT in response to secreted factors by MMA-treated fibroblasts. a. Immunoblots of A549 and A375 tumor cells after 5 days of treatment by the supernatant of conditioned media from vehicle or MMA-treated MRC-5 (for A549) or BJ (for A375) fibroblasts after EVs isolation. **b**. Immunoblots of A549 cells treated with veh-EVs and MMA-EVs for 5 days, then co-cultured with or without untreated MRC-5 fibroblasts for 5 days.

S Figure 4

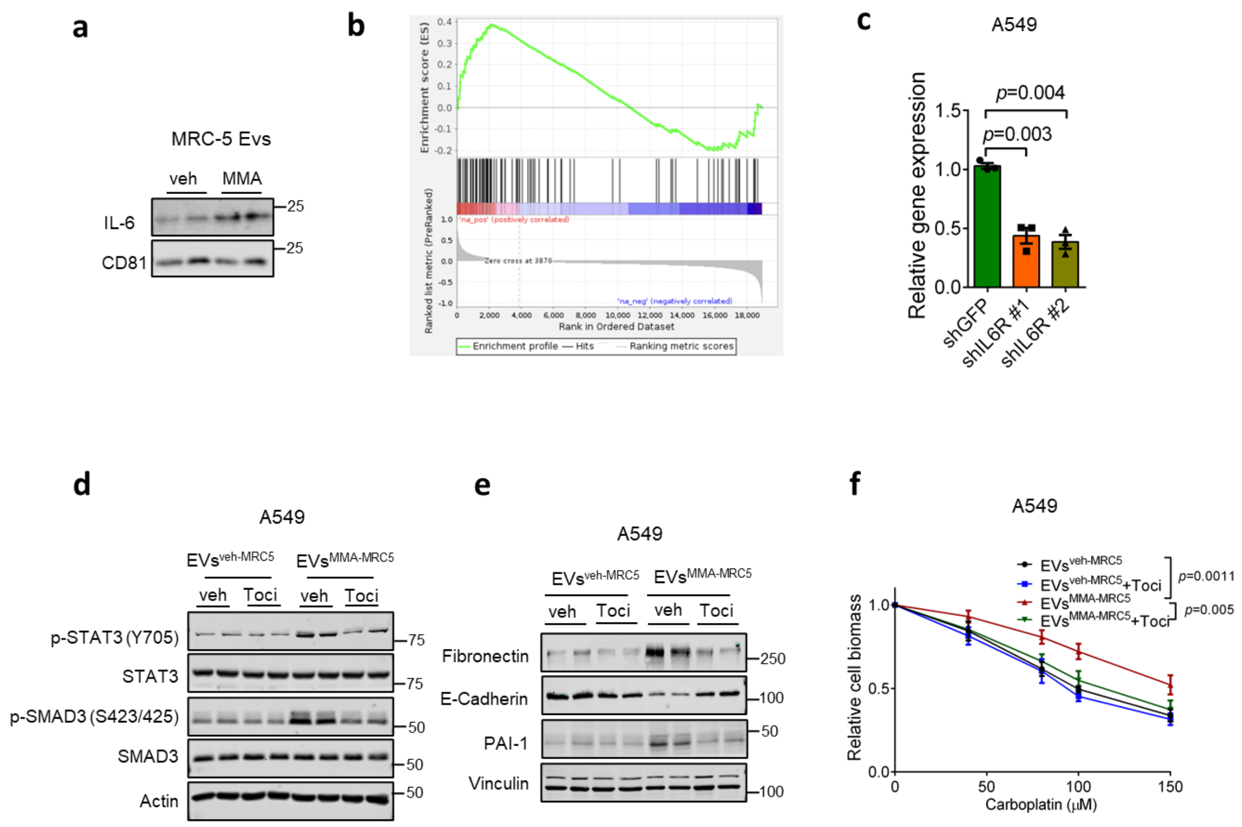

Supplementary Figure 4. IL-6R activation is essential for EMT induction of acquisition of aggressive traits in tumor cells. a. Immunoblots showing levels of IL-6 in veh-EVs and MMA-EVs. **b**. Cells were ranked along an epithelial-to-mesenchymal continuum based on the MES Ratio, which is defined as the log10 transform of the mean imputed expression ratio of VIM and EPCAM for each cell. Gene set enrichment analyses (GSEA) was then performed for genes ranked by their correlation with this continuum using Hallmark Gene Set in the Molecular Signature Database (REF: http://www.gsea-msigdb.org/gsea/msigdb/collections.jsp). Running enrichment plot shown for IL6-JAK-STAT pathway. **c.** RT-qPCR measurement of IL-6R mRNA in A549 cells after 3 days of shIL-6R knockdown (n=3 independent experiments, mean ± SEM, two-sided paired t-test). **d.** Immunoblots showing signaling activation in A549 cells 3 hours after treatment by veh-EVs or MMA-EVs from MRC-5 fibroblasts, with or without IL-6R inhibitor Tocilizumab. **e, f.** Pro-aggressive properties of A549 tumor cells 5 days after treatment by veh-EVs or MMA-EVs from MRC-5 fibroblasts, with or without IL-6R inhibitor Tocilizumab, evaluated by immunoblots measuring EMT marker expression (**e**) and carboplatin resistance assay (f; n=3 independent experiments, mean ± SEM, two-way ANOVA) (**f**).

S Figure 5

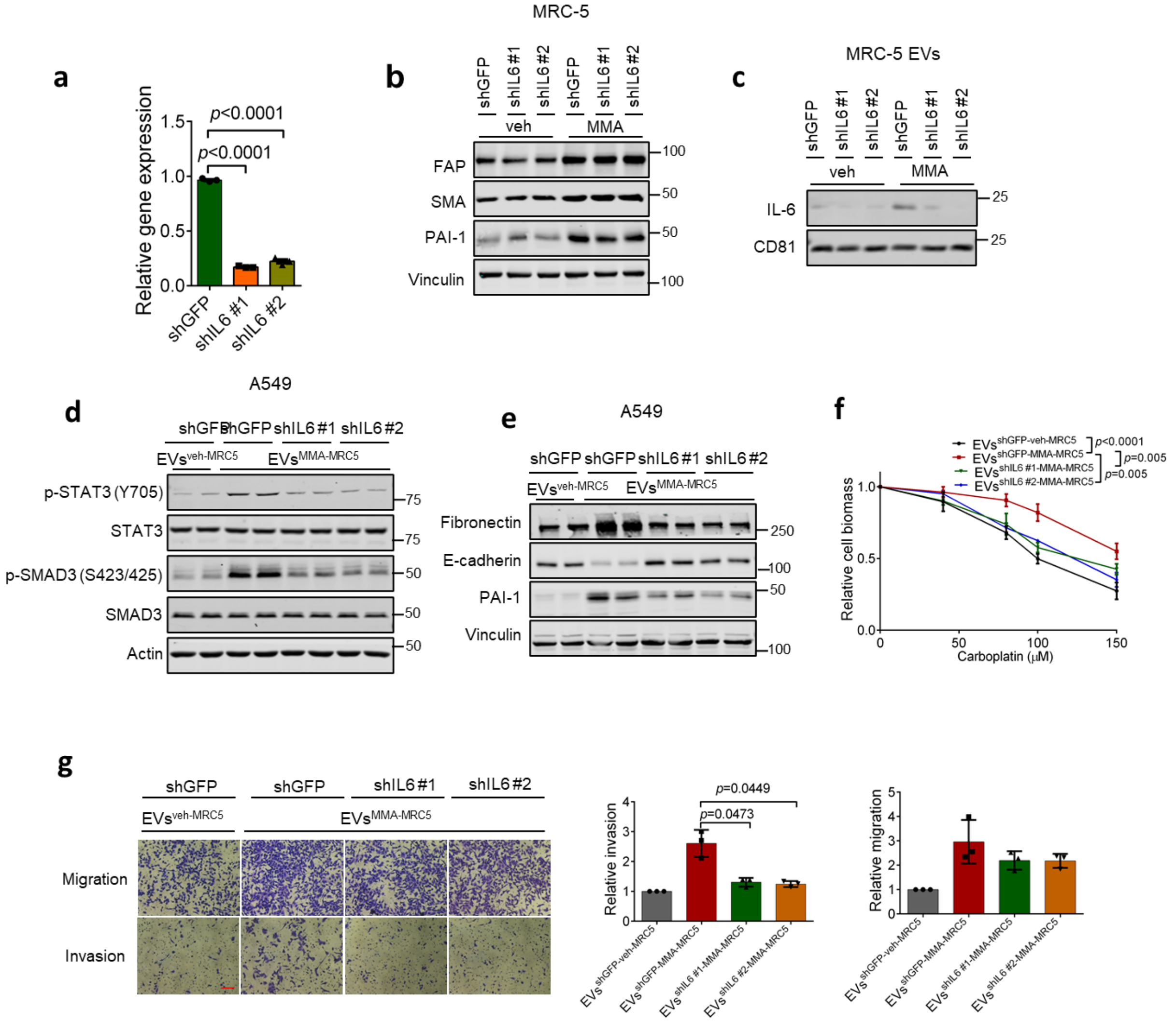

Supplementary Figure 5. IL6 is necessary but not sufficient to induce EMT and pro-aggressive traits in tumor cells. a. RT-qPCR measurement of IL-6 mRNA in MRC-5 fibroblasts 5 days after infection (n=3 independent experiments, mean ± SEM, two-sided paired t-test). **b.** Immunoblots of CAF markers in MRC-5 fibroblasts with or without sh*IL6* knockdown, with or without 1mM MMA treatment. **c.** Immunoblots of IL-6 amount in EVs isolated from MRC-5 fibroblasts with or without sh*IL6* knockdown, with or without 1mM MMA treatment. **d.** Immunoblots measuring signaling activation in A549 cells 3 hours after treatment with EVs from MRC-5 fibroblasts with or without sh*IL6* knockdown and MMA treatment. **e, f, g.** Pro-aggressive traits of A549 cells 3 days after treatment with veh-EVs or MMA-EVs from MRC-5 fibroblasts with sh*GFP* or sh*IL6* knockdown and MMA treatment, evaluated by immunoblots measuring EMT marker expression (**e**), carboplatin resistance assay (**f;** n=3 independent experiments, mean ± SEM, two-way ANOVA), and invasion and migration transwell assays (**g;** red scale bar indicates 100μM, n=3 independent experiments, mean ± SEM, two-sided paired t-test).

S Figure 6

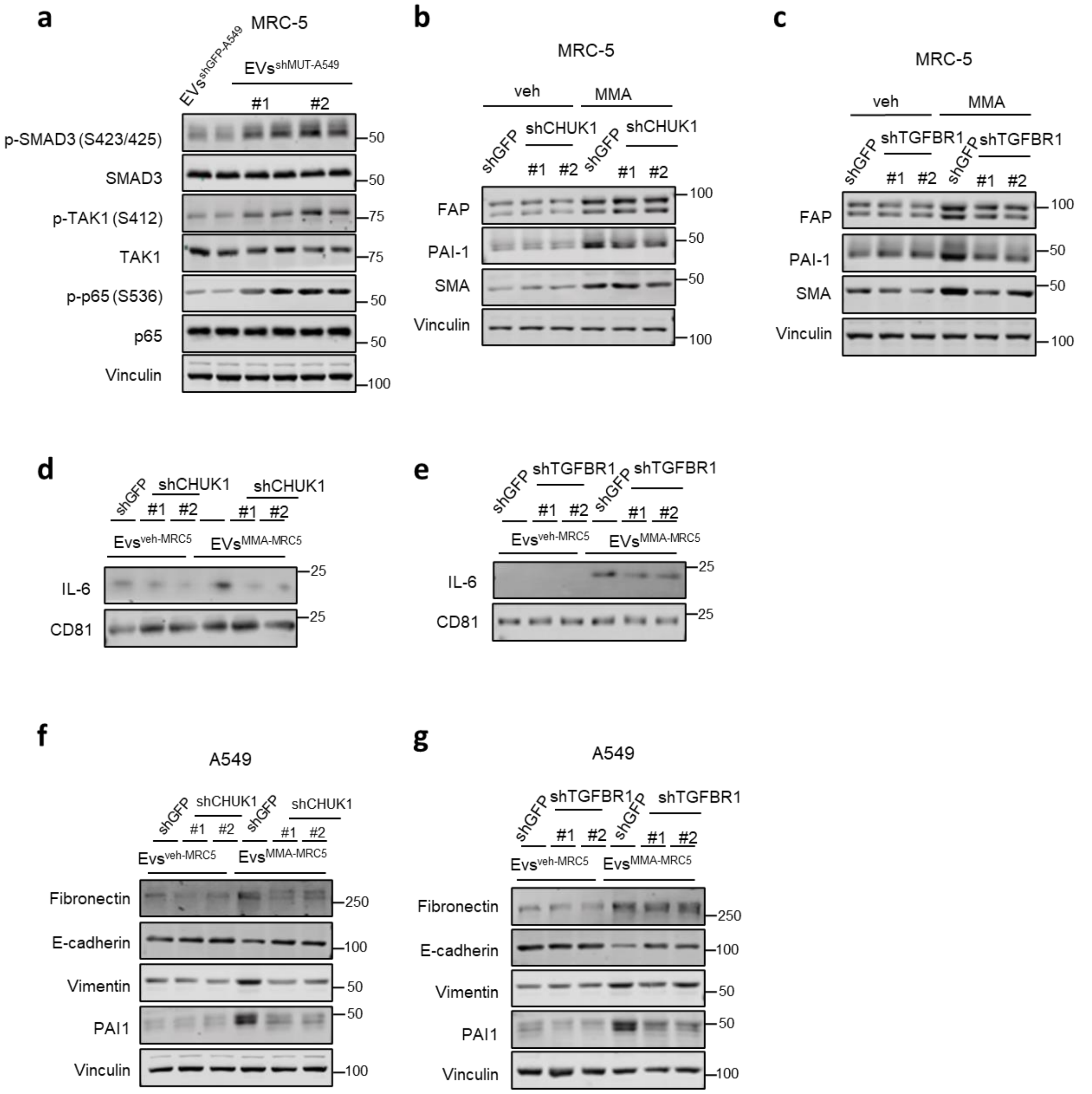

Supplementary Figure 6. TGFβ receptor activation is required for MMA to activate CAF markers, and IKK is required for MMA-induced IL-6 secretion in EVs. a. Immunoblots measuring signaling activation in MRC-5 fibroblasts treated with A549 tumor cell-derived EVs for 3 hours. **b, c.** Immunoblots measuring CAF markers in *GFP* and *CHUK1*(**b**) or *TGFBR1* (**c**) knocked-down MRC-5 fibroblasts treated with MMA for 5 days. **d, e**. Immunoblots measuring IL-6 in the EVs isolated from MMA-treated MRC-5 fibroblasts with *CHUK1* or *TGFBR1* knocked down. **f, g**. Immunoblots measuring EMT markers in A549 tumor cells treated with EVs isolated from MRC-5 fibroblasts for 3 days.

S Figure 7

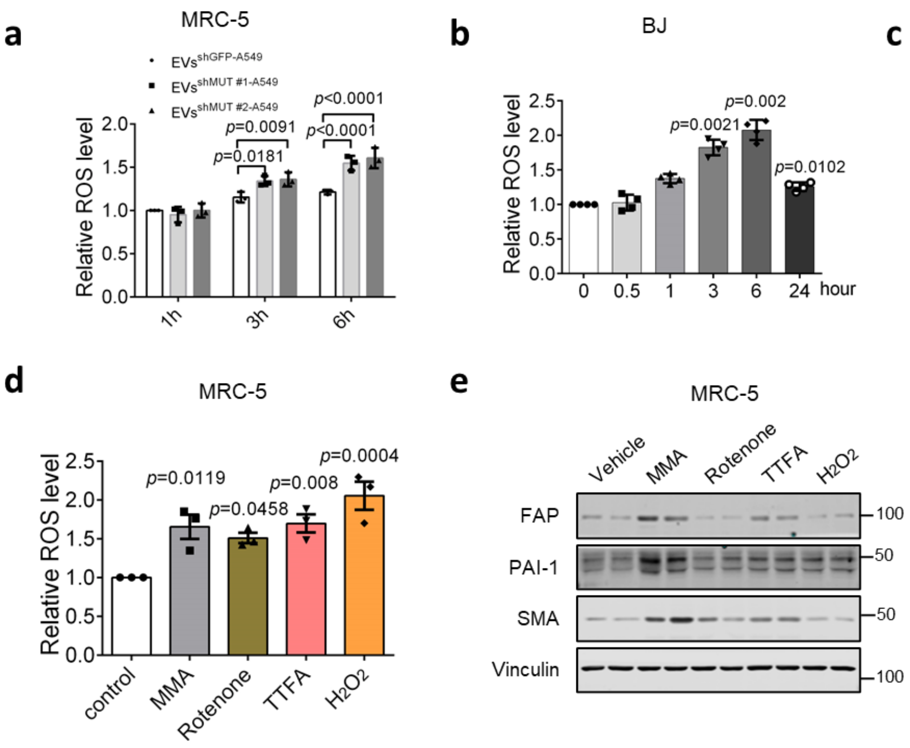

Supplementary Figure 7. ROS is required for MMA activation of fibroblasts driving metastasis. a. ROS levels in MRC-5 fibroblasts after treatment by EVs from A549 cells with sh*GFP* or sh*MUT* knockdown (n=3, mean ± SEM, one-way ANOVA). **b**. ROS levels and (**c**) MDA levels in BJ fibroblasts after 1mM of MMA treatment (n=4 independent experiments for ROS measurement, mean ± SEM, one-way ANOVA; n=3 independent experiments for MDA measurement, mean ± SEM, two-sided paired t-test). **d, e**. ROS levels (n=4 independent experiments, mean ± SEM, one-way ANOVA) (**d**) and immunoblots measuring CAF markers (**e**) of MRC-5 fibroblasts after treatment by different ROS inducers (1mM MMA, 100nM rotenone, 100μM TTFA, 10μM H_2O_2) for 6 hours (**d**) or 5 days (**e**).
