# Supplementary material for: Tumor-produced and aging-associated oncometabolite methylmalonic acid promotes cancer-associated fibroblast activation to drive metastatic progression
Source: Nat Commun. 2022 Oct 20;13:6239. doi: 10.1038/s41467-022-33862-0 (PMC9584945; doi:10.1038/s41467-022-33862-0)
Supplement: Supplementary file 3 — Reporting Summary [file 41467_2022_33862_MOESM3_ESM.pdf]

nature portfolio

Corresponding author(s): John Blenis

Last updated by author(s): Sep 15, 2022

Reporting Summary

Nature Portfolio wishes to improve the reproducibility of the work that we publish. This form provides structure for consistency and transparency in reporting. For further information on Nature Portfolio policies, see our Editorial Policies and the Editorial Policy Checklist.

Statistics

For all statistical analyses, confirm that the following items are present in the figure legend, table legend, main text, or Methods section.

n/a	Confirmed	
☐	☒	The exact sample size (*n*) for each experimental group/condition, given as a discrete number and unit of measurement
☐	☒	A statement on whether measurements were taken from distinct samples or whether the same sample was measured repeatedly
☐	☒	The statistical test(s) used AND whether they are one- or two-sided<br>*Only common tests should be described solely by name; describe more complex techniques in the Methods section.*
☒	☐	A description of all covariates tested
☒	☐	A description of any assumptions or corrections, such as tests of normality and adjustment for multiple comparisons
☐	☒	A full description of the statistical parameters including central tendency (e.g. means) or other basic estimates (e.g. regression coefficient) AND variation (e.g. standard deviation) or associated estimates of uncertainty (e.g. confidence intervals)
☐	☒	For null hypothesis testing, the test statistic (e.g. F, t, r) with confidence intervals, effect sizes, degrees of freedom and P value noted<br>*Give P values as exact values whenever suitable.*
☒	☐	For Bayesian analysis, information on the choice of priors and Markov chain Monte Carlo settings
☒	☐	For hierarchical and complex designs, identification of the appropriate level for tests and full reporting of outcomes
☒	☐	Estimates of effect sizes (e.g. Cohen's d, Pearson's r), indicating how they were calculated

Our web collection on statistics for biologists contains articles on many of the points above.

Software and code

Policy information about availability of computer code

Data collection	For targeted metabolomics peak areas from the total ion current for each metabolite SRM transition were integrated using MultiQuant v2.0 software (AB/SCIEX). For qPCR, QuantStudio Real Time PCR software v1.3 (Life Technologies) was utilized. For FACS BD Accuri C6 software v1.0.264.21 was utilized. For xenografted cells in mice luminescence was measured and quantified using the Living Image Software v4.5 (Perkin-Elmer).
Data analysis	All data analyses are explained in detail in the online methods. General data analyses were performed using Microsoft Excel 2013 and GraphPad Prism7. For targeted metabolomics statistical analysis of the data was carried out using MetaboAnalyst v4.0, a free online software for the analysis of metabolomic experiments (www.metaboanalyst.ca). For RNA-seq: After trimming the adaptors with Cutadapt (version1.18)(https://cutadapt.readthedocs.io/en/v1.18/), RNA reads were aligned and mapped to the GRCh38 human reference genome by STAR (Version2.5.2) (https://github.com/alexdobin/STAR) (Dobin et al., 2013), and transcriptome reconstruction was performed by Cufflinks (Version 2.1.1) (http://cole-trapnell-lab.github.io/cufflinks/). The abundance of transcripts was measured with Cufflinks in Fragments Per Kilobase of exon model per Million mapped reads (FPKM) (Trapnell et al., 2013; Trapnell et al., 2010). Gene expression profiles were constructed for differential expression, cluster, and principle component analyses with the DESeq2 package (https://bioconductor.org/packages/release/bioc/html/DESeq2.html) (Love et al., 2014). For differential expression analysis, pairwise comparisons between two or more groups using parametric tests where read-counts follow a negative binomial distribution with a gene-specific dispersion parameter. Corrected p-values were calculated based on the Benjamini-Hochberg method to adjusted for multiple testing. For proteomics analysis: MS/MS spectra were matched to peptide sequences using COMET (version 2019.01 rev. 5) (Eng et al., 2013) and a composite database containing the 20,415 Uniprot reviewed canonical predicted human protein sequences (http://uniprot.org, downloaded 5/1/2019) and its reversed complement.

For manuscripts utilizing custom algorithms or software that are central to the research but not yet described in published literature, software must be made available to editors and reviewers. We strongly encourage code deposition in a community repository (e.g. GitHub). See the Nature Portfolio guidelines for submitting code & software for further information.

Data

Policy information about availability of data

All manuscripts must include a data availability statement. This statement should provide the following information, where applicable:

- Accession codes, unique identifiers, or web links for publicly available datasets
- A description of any restrictions on data availability
- For clinical datasets or third party data, please ensure that the statement adheres to our policy

All data generated or analysed during this study are included in this published article (and its supplementary information files).

Field-specific reporting

Please select the one below that is the best fit for your research. If you are not sure, read the appropriate sections before making your selection.

[x] Life sciences [ ] Behavioural & social sciences [ ] Ecological, evolutionary & environmental sciences

For a reference copy of the document with all sections, see nature.com/documents/nr-reporting-summary-flat.pdf

Life sciences study design

All studies must disclose on these points even when the disclosure is negative.

Sample size	No sample size calculations were done. For in vivo studies, sample sizes were determined based on our previous experiments. In our experience, n = 7-10 mice per group is sufficient to detect meaningful biological differences with good reproducibility. Treatment/analysis n refers to biologically independent samples as individual mice or human donors. The vast majority of the experiments outlined in this manuscript utilize cell-based systems, where conditions are standard and extreme variability is decreased as a result. Thus, we utilized at least 3 independent samples for each nominal variable. This number of independent experiments is a standard sample size to accurately detect differences in cell biology and molecular biology experiments (such as Napolitano et al. Nature 2020. https://doi.org/10.1038/s41586-020-2444-0.) Western blots are shown as representative images, however they were reproduced with at least 3 independent samples. Figure legends indicate when n refers to independent biological samples versus samples from independent experiments.
Data exclusions	No data was excluded
Replication	Experiments were repeated multiple times with similar results as indicated in the figure legends. All attempts to replicate the data were successful. The core experiments were replicated by at least two people independently and in multiple cell lines to insure reproducibility and validity of the experiments.
Randomization	Mice obtained from the vendor were randomly split into experimental groups before administering xenografts. No randomization was done for cell culture experiments, same plate of cells were used to set up the treatment groups in each experiment.
Blinding	Blinding was not performed for the in vitro and in vivo experiments. The investigators needed to know the treatment groups in order to perform the study, and the data analyses were based on objectively measurable data.

Reporting for specific materials, systems and methods

We require information from authors about some types of materials, experimental systems and methods used in many studies. Here, indicate whether each material, system or method listed is relevant to your study. If you are not sure if a list item applies to your research, read the appropriate section before selecting a response.

Materials & experimental systems

n/a	Involved in the study	
[ ]	[x]	Antibodies
[ ]	[x]	Eukaryotic cell lines
[x]	[ ]	Palaeontology and archaeology
[ ]	[x]	Animals and other organisms
[x]	[ ]	Human research participants
[x]	[ ]	Clinical data
[x]	[ ]	Dual use research of concern

Methods

n/a	Involved in the study	
[x]	[ ]	ChIP-seq
[x]	[ ]	Flow cytometry
[x]	[ ]	MRI-based neuroimaging

Antibodies

Antibodies used

Proteins of interest were probed with specific antibodies (listed as 'target protein' (catalog no. - vendor, dilution factor): FAP (66562s – Cell Signaling Technology, 1:1000), SMA (ab5694 – Abcam, 1:1000), CAV-1(ab2910 – Abcam, 1:1000), PAI-1 (612024 – BD, 1:1000), PCCA (ab187686 – Abcam, 1:2000), Vinculin (V9264 – Sigma Aldrich, 1:5000), MMP-2 (4022S – Cell Signaling Technology 1:1000), MMP-14 (ab51074 – Abcam, 1:1000), MMP-13 (ab39012 – Abcam, 1:1000), CTGF (ab6992 – Abcam, 1:500), E-Cadherin (610181 – BD, 1:1000), Fibronectin (ab2413 – Abcam, 1:10,000), Vimentin (5741S – Cell Signaling Technology, 1:2000), Snail (3879S – Cell Signaling Technology, 1:1000), p-JAK2(Y1007/1008) (3771 – Cell Signaling Technology, 1:500), JAK2 (3230 – Cell Signaling Technology, 1:1000), p-Stat3 Y705 (ab76315 – Abcam, 1:1000), p-Stat3 S727 (9136S – Cell Signaling Technology, 1:1000), Stat3 (9139S – Cell Signaling Technology, 1:1000), p-Smad3 S423/425 (ab52903 – Abcam, 1:1000), Smad3 (9523S – Cell Signaling Technology, 1:1000), IL-6 (12153S – Cell Signaling Technology, 1:1000), CD81 (56039S – Cell Signaling Technology, 1:1000), CD9 (ab223052 – Abcam, 1:500), Flotillin-1 (610820 – BD, 1:1000), GM130 (610823 – BD, 1:1000), Lamin (4777 – Cell Signaling Technology, 1:1000), Calnexin (ab112995 – Abcam, 1:1000), β-Actin (4967 – Cell Signaling Technology, 1:2000), p-Smad2 S465/467 (3108S – Cell Signaling Technology, 1:1000), Smad2 (3103S, Cell Signaling Technology, 1:1000), p65 (8242S – Cell Signaling Technology, 1:1000), p-p65 S536 (3036S – Cell Signaling Technology, 1:1000), p-IκBα (Ser32/36) (9246S – Cell Signaling Technology, 1:1000), IκBα (9242S – Cell Signaling Technology, 1:1000), TAK1 (5206S – Cell Signaling Technology, 1:1000), p-TAK1 S412 (9339S – Cell Signaling Technology, 1:1000). anti-rabbit (NA934 - GE Healthcare, 1:10,000), anti-mouse (NA931 – GE Healthcare, 1:10,000) or anti-goat (AP180P - Millipore, 1:10,000) immunoglobulin for 1h at room temperature, and signals developed using Amersham ECL detection system (GE Healthcare), or they were incubated with the appropriate donkey anti-rabbit Alexa Fluor 488 (A-21206 – Thermo Fisher Scientific, 1:10,000) or donkey anti-mouse Alexa Fluor 555 (A31570 – Thermo Fisher Scientific, 1:10,000).

Validation

All antibodies used are commercially available and were tested by the manufacturer. They are standard tools used in the field, and have been previously validated and characterized by multiple labs. More information about each antibody and links to product citations can be found on manufacturers' websites.

FAP (66562s – Cell Signaling Technology, 1:1000): https://www.cellsignal.com/products/primary-antibodies/fap-e1v9v-rabbit-mab/66562
SMA (ab5694 – Abcam, 1:1000): https://www.abcam.com/alpha-smooth-muscle-actin-antibody-ab5694.html
CAV-1(ab2910 – Abcam, 1:1000): https://www.abcam.com/Caveolin-1-antibody-Caveolae-Marker-ab2910.html?gclsrc=aw.ds|aw.ds&gclid=CjwKCAjwiY6MBhBqEiwARFSCPhalrsGmfv2WKI3wd0pLPVgoUDk7_zSmObxMflIoTnqVH2rTBYav_RoCoz8QAvD_BwE
PAI-1 (612024 – BD, 1:1000): https://www.bdbiosciences.com/en-us/products/reagents/western-blotting-and-molecular-reagents/western-blot-reagents/purified-mouse-anti-pai-1.612025
Vinculin (V9264 – Sigma Aldrich, 1:5000): https://www.sigmaaldrich.com/US/en/product/SIGMA/V9131?gclid=CjwKCAjwiY6MBhBqEiwARFSCPrHmL2zSRgIAZg6WkVynyClGsz6b4rCx0r44Cesxqb1MFyn332LiuRoCuy0QAvD_BwE
MMP-2 (4022S – Cell Signaling Technology 1:1000): https://www.cellsignal.com/products/primary-antibodies/mmp-2-antibody/4022
MMP-14 (ab51074 – Abcam, 1:1000): https://www.abcam.com/mmp14-antibody-ep1264y-ab51074.html
MMP-13 (ab39012 – Abcam, 1:1000): https://www.abcam.com/mmp14-antibody-ep1264y-ab51074.html
CTGF (ab6992 – Abcam, 1:500): https://www.abcam.com/ctgf-antibody-ab6992.html
E-Cadherin (610181 – BD, 1:1000): https://www.bdbiosciences.com/en-us/products/reagents/microscopy-imaging-reagents/immunofluorescence-reagents/purified-mouse-anti-e-cadherin.610181
Fibronectin (ab2413 – Abcam, 1:10,000): https://www.abcam.com/fibronectin-antibody-ab2413.html
Vimentin (5741S – Cell Signaling Technology, 1:2000): https://www.cellsignal.com/products/primary-antibodies/vimentin-d21h3-xp-rabbit-mab/5741
Snail (3879S – Cell Signaling Technology, 1:1000): https://www.cellsignal.com/products/primary-antibodies/snail-c15d3-rabbit-mab/3879
p-JAK2(Y1007/1008) (3771 – Cell Signaling Technology, 1:500): https://www.cellsignal.com/products/primary-antibodies/phospho-jak2-tyr1007-1008-antibody/3771
JAK2 (3230 – Cell Signaling Technology, 1:1000): https://www.cellsignal.com/products/primary-antibodies/jak2-d2e12-xp-rabbit-mab/3230
p-Stat3 Y705 (ab76315 – Abcam, 1:1000): https://www.abcam.com/stat3-phospho-y705-antibody-ep2147y-ab76315.html
p-Stat3 S727 (9136S – Cell Signaling Technology, 1:1000): https://www.cellsignal.com/products/primary-antibodies/phospho-stat3-ser727-6e4-mouse-mab/9136
Stat3 (9139S – Cell Signaling Technology, 1:1000): https://www.cellsignal.com/products/primary-antibodies/stat3-124h6-mouse-mab/9139
p-Smad3 S423/425 (ab52903 – Abcam, 1:1000): https://www.abcam.com/smad3-phospho-s423--s425-antibody-ep823y-ab52903.html
Smad3 (9523S – Cell Signaling Technology, 1:1000): https://www.cellsignal.com/products/primary-antibodies/smad3-c67h9-rabbit-mab/9523
IL-6 (12153S – Cell Signaling Technology, 1:1000): https://www.cellsignal.com/products/primary-antibodies/il-6-d3k2n-rabbit-mab/12153
CD81 (56039S – Cell Signaling Technology, 1:1000): https://www.cellsignal.com/products/primary-antibodies/cd81-d3n2d-rabbit-mab/56039?site-search-type=Products&N=4294956287&Ntt=56039&fromPage=plp&_requestid=1311536
CD9 (ab223052 – Abcam, 1:500): https://www.abcam.com/cd9-antibody-ab223052.html
Flotillin-1 (610820 – BD, 1:1000): https://www.bdbiosciences.com/en-us/products/reagents/microscopy-imaging-reagents/immunofluorescence-reagents/purified-mouse-anti-flotillin-1.610820
GM130 (610823 – BD, 1:1000): https://www.bdbiosciences.com/en-us/products/reagents/microscopy-imaging-reagents/immunofluorescence-reagents/purified-mouse-anti-gm130.610823
Lamin (4777 – Cell Signaling Technology, 1:1000): https://www.cellsignal.com/products/primary-antibodies/lamin-a-c-4c11-mouse-mab/4777
Calnexin (ab112995 – Abcam, 1:1000): https://www.abcam.com/calnexin-antibody-6f12be10-ab112995.html
β-Actin (4967 – Cell Signaling Technology, 1:2000): https://www.cellsignal.com/products/primary-antibodies/b-actin-antibody/4967

p-Smad2 S465/467 (3108S – Cell Signaling Technology, 1:1000): https://www.cellsignal.com/products/primary-antibodies/phospho-smad2-ser465-467-138d4-rabbit-mab/3108
Smad2 (3103S, Cell Signaling Technology, 1:1000): https://www.cellsignal.com/products/primary-antibodies/smad2-l16d3-mouse-mab/3103
p65 (8242S – Cell Signaling Technology, 1:1000): https://www.cellsignal.com/products/primary-antibodies/nf-kb-p65-d14e12-xp-rabbit-mab/8242
p-p65 S536 (3036S – Cell Signaling Technology, 1:1000): https://www.cellsignal.com/products/primary-antibodies/phospho-nf-kb-p65-ser536-7f1-mouse-mab/3036
p-IκBα (Ser32/36) (9246S – Cell Signaling Technology, 1:1000): https://www.cellsignal.com/products/primary-antibodies/phospho-ikba-ser32-36-5a5-mouse-mab/9246
IκBα (9242S – Cell Signaling Technology, 1:1000): https://www.cellsignal.com/products/primary-antibodies/ikba-antibody/9242
TAK1 (5206S – Cell Signaling Technology, 1:1000): https://www.cellsignal.com/products/primary-antibodies/tak1-d94d7-rabbit-mab/5206
p-TAK1 S412 (9339S – Cell Signaling Technology, 1:1000): https://www.cellsignal.com/products/primary-antibodies/phospho-tak1-ser412-antibody/9339

Eukaryotic cell lines

Policy information about cell lines

Cell line source(s)	A549 cells (non-small cell lung cancer; CCL-185), A375 human melanoma cells (CRL-1619), MRC-5 human lung fibroblasts (CCL-171) and BJ foreskin fibroblasts (CRL-2522) were obtained from the American Type Culture Collection (ATCC). HEK293T cells were obtained from GenHunter
Authentication	ATCC utilizes multiple methods - morphology, karyotyping, and PCR based approaches - to authenticate the cell lines it maintains. No further authentication was performed by the authors of this manuscript.
Mycoplasma contamination	All cell lines were routinely tested for mycoplasma and were at all times mycoplasma negative.
Commonly misidentified lines (See ICLAC register)	No commonly misidentified lines were used in this study

Animals and other organisms

Policy information about studies involving animals; ARRIVE guidelines recommended for reporting animal research

Laboratory animals	Female nu/nu athymic mice (Envigo) were purchased at the age of 4-6 weeks, and the experiments were started 7-10 days after the mice were received at the Weill Cornell Medicine Belfer Research Building Vivarium. Experimental groups of 7-10 mice were created randomly and mice were group housed, maximum 5, in standard cages with unrestricted water and food, PicoLab Rodent Diet 5053 (Labdiet, Purina) containing 20% protein and 5% fat. Only deviation from the standard housing was for animals that received MMA in their drinking water as described in “Orthotopic Xenograft Experiments in Mice” section. Animal husbandry was carried out by the vivarium technical staff in a human xenograft designated area following animal biosafety level-2 procedures. The room was maintained at 21-23°C and a 12h light-dark cycle. The mice were maintained in compliance to Weill Cornell Medicine Institutional Animal Care and Use Committee protocols.
Wild animals	No wild animals were used in this study.
Field-collected samples	No field-collected samples were used in this study.
Ethics oversight	The mice were maintained at Weill Cornell Medicine in compliance to Weill Cornell Medicine Institutional Animal Care and Use Committee protocols.

Note that full information on the approval of the study protocol must also be provided in the manuscript.
